# Notes and News

**Published:** 1896-01-18

**Authors:** 


### Notes and News.

(Collected and Communicated.)

Quarantine at Spanish ports has been ordered on ships arriving from Casabianca and Mazagan, where cholera has appeared.

We learn that Mr. W. E. Hartopp has been appointed secretary to the Grosvenor Hospital for Women and Children, in succession to Mr. Harvey.

A new pavilion has just been added to the City Hospital, Aberdeen. It will accommodate 32 patients in two wards. The furnishing and fittings were much admired by the company assembled for the opening ceremony, presided over by the Lord Provost.

The speeches at the dinner of the Society of Public Analysts were not markedly concerned with topics connected with the cult. The political situation was too strong a rival. Nevertheless, Dr. Voelcker found opportunity to say that a special training was necessary to the following of the profession of a public analyst, whilst Dr. Thorne Thorne assured those present that the department at Whitehall aimed at raising the standard and status of public analysts. Amongst those present were: Professor Sharpe, Mr. A. Vernon Harcourt, president of the Chemical Society, Dr. Dupre, and Mr. Otto Helmer.

The Christmas gift of £200, which our contemporary, *The Gentlewoman*, announced some time since would be distributed amongst five charities, to be chosen by votes on the part of readers of the paper, has now been awarded. The competition, it will be remembered, was limited to institutions in Ireland, Scotland, Wales, Yorkshire, and Devonshire. The poll was closed on December 31st, up to which day a total of 17,416 votes were received. Ireland heads the poll with 11,122 votes for the Orthopædic Hospital, Great Brunswick Street, Dublin; Scotland comes next with 1,201 votes for the Orphanage, Aberlour, Strathspey; and for Wales the Llanelly Nursing Institution, Carmarthenshire, secured 844 votes. The Victoria Children's Hospital, Hull, and the Devon and Exeter Hospital, Exeter, close the list with 626 and 80 votes respectively. The three first-named charities receive £50, the two last £25 apiece. If each voter expended sixpence on a copy of *The Gentlewoman*, this competition has yielded its proprietors an apparent profit of upwards of £230.

A Hungarian lady, who was recently returning from treatment at the Pasteur Institute at Budapest for a bite by a mad dog, was seized with hydrophobia in the train, and removed to hospital.

Amongst the inhabitants of Johannesburg who have been arrested as leaders of the Reform Committee is Mr. W. St. John Carr, chairman of the Hospital Committee.

Paul Verlaine, the French poet, whose death was announced last week, was a frequent inmate of hospitals, and he has related some experiences in his book entitled, "My Hospitals," which was published some five years ago.

The Commissioners in Lunacy have replied to the deputation on behalf of Miss Lanchester that they cannot proceed against Dr. Blandford for issuing a false certificate of lunacy. They admit Dr. Blandford made a mistake, but there was no evidence of corrupt motive.

The hospital ship Coromandel, which we described in our columns before she left the Thames for Ashanti, has found plenty to do in her new quarters. The sick list has numbered as many as sixty at one time. The ailments were reported as slight, and the patients as all doing well.

We are not surprised to see that the statement made in a contemporary medical journal, on the authority of Dr. Chapman, has been contradicted by him. This statement was to the effect that cider is responsible for a very large proportion of the insanity treated in the Burghill Asylum, Hereford. Cider has hitherto been regarded as a healthy beverage, and it is somewhat alarming to learn that it possessed qualities liable to induce insanity. Dr. Chapman repudiates the interpretation placed upon his words, however, and we can once more set at rest any doubt that the inhabitants of cider-making countries are really in greater danger from partaking of this form of alcohol than any other however potent.

There can be no doubt that there is a right and a wrong way of setting about securing funds for charitable objects, or we should not see such varied success in like fields of operation. Object lessons always prove useful to the wise, and therefore we hold up the recent success at Ayr as an example. A fancy ball was organised on behalf of the County Hospital. Not only were patrons invited to buy tickets—thus, as is the usual custom, actually receiving some return for their so-called charity—but at Ayr contributions in kind and in money towards the expenses of the ball were solicited. The response was so hearty that £329 was collected, whilst gifts of poultry, game, aerated waters, and other provisions considerably lessened the cost of the supper. Three hundred and eleven pounds was secured through the sale of tickets, and when all expenses were deducted there remained the handsome sum of £500, which was handed over to the hospital. Everyone seems to have combined to assist, in reducing expenses and otherwise, and thus interest was stimulated, and the splendid result secured. Where can we find a record to beat this one of a fancy ball at Ayr? We hope the example will be emulated where funds are needed with as good or even a better result.

